# Regulation of Signaling and Metabolism by Lipin-mediated Phosphatidic Acid Phosphohydrolase Activity

**DOI:** 10.3390/biom10101386

**Published:** 2020-09-29

**Authors:** Andrew J. Lutkewitte, Brian N. Finck

**Affiliations:** Center for Human Nutrition, Division of Geriatrics and Nutritional Sciences, Department of Medicine, Washington University School of Medicine, Euclid Avenue, Campus Box 8031, St. Louis, MO 63110, USA; alutkew@wustl.edu

**Keywords:** phosphatidic acid, diacylglycerol, lipin, signaling

## Abstract

Phosphatidic acid (PA) is a glycerophospholipid intermediate in the triglyceride synthesis pathway that has incredibly important structural functions as a component of cell membranes and dynamic effects on intracellular and intercellular signaling pathways. Although there are many pathways to synthesize and degrade PA, a family of PA phosphohydrolases (lipin family proteins) that generate diacylglycerol constitute the primary pathway for PA incorporation into triglycerides. Previously, it was believed that the pool of PA used to synthesize triglyceride was distinct, compartmentalized, and did not widely intersect with signaling pathways. However, we now know that modulating the activity of lipin 1 has profound effects on signaling in a variety of cell types. Indeed, in most tissues except adipose tissue, lipin-mediated PA phosphohydrolase activity is far from limiting for normal rates of triglyceride synthesis, but rather impacts critical signaling cascades that control cellular homeostasis. In this review, we will discuss how lipin-mediated control of PA concentrations regulates metabolism and signaling in mammalian organisms.

## 1. Introduction

**Figure 1:**
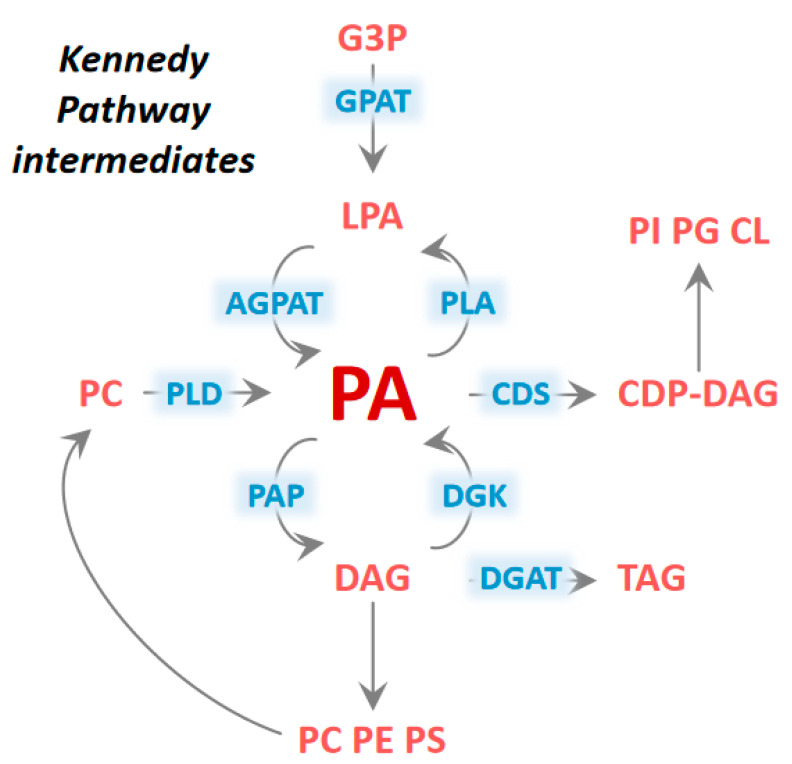
Phosphatidic acid as a central component in the Kennedy Pathway of lipid synthesis. Phosphatidic acid (PA) can be synthesized from and converted to numerus glycerophospholipids involved in membrane formation, cell signaling, lipid storage, and many others. Enzyme abbreviations in blue: glycerol-3-phosphate acyltransferase (GPAT), 1-acylglycerol-3-phosphate O-acyltransferase (AGPAT), phospholipase A (PLA), phospholipase D (PLD), cytidine diphosphate diacylglycerol Synthase (CDS), diacylglycerol kinase (DGK), phosphatidic acid phosphatase (PAP), diacylglycerol O-acyltransferase (DGAT). Glycerophospholipids and derivatives abbreviations in red: glycerol-3-phosphate (G3P), lysophosphatidic acid (LPA), phosphatidic acid (PA), phosphatidylcholine (PC), cytidine diphosphate diacylglycerol (CDP-DAG), phosphatidylinositol (PI), phosphatidylglycerol (PG), cardiolipin (CL), diacylglycerol (DAG), triacylglycerol (TAG), phosphatidylcholine (PC), phosphatidylethanolamine (PE), phosphatidylserine (PS).

Foundational work many decades ago by the laboratory of Dr. Eugene Kennedy defined the four sequential enzymatic steps by which three fatty acyl groups were esterified onto the glycerol-3-phosphate backbone to synthesize triglyceride [1]. The penultimate step in this pathway, the dephosphorylation of phosphatidic acid (PA) to form diacylglycerol (DAG), is catalyzed by Mg^2+^-dependent PA phosphohydrolase (PAP) enzymes; an enzymatic activity first quantified in 1957 [2]. This lipid had been measured in plants, but at that time, the existence of PA in *Animalia* was controversial. It is now known that PA is maintained at picomolar concentrations in most cells and that this glycerophospholipid constitutes a critical branching-point in the Kennedy Pathway (Figure 1). PA is the precursor of cytidine diphosphate diacylglycerol (CDP-DAG) used to make several phospholipids including phosphatidylglycerol and phosphatidylinositol, while DAG is the substrate for synthesis of other abundant phospholipids like phosphatidylcholine and phosphatidylethanolamine. Although the elegant studies of Kennedy described PAP activity in chicken liver at a biochemical level in 1957 [2], the cloning of the genes that encode proteins with PAP catalytic activity would require almost 50 years of additional study [3,4]

Convergent lines of research in multiple model organisms and serendipitous findings with freezer-archived samples would eventually lead to the identification of the mammalian lipin family of proteins as PAP enzymes [5]. In 2006, the lab of George Carman reported that the yeast *Pah* protein catalyzed the long sought Mg^2+^-dependent PAP activity in yeast [3]. This protein was the yeast homolog of the mammalian lipin family of proteins that were identified by Dr. Karen Reue’s group in 2001 [4], but at that time they had no known molecular function. Han and colleagues demonstrated that, like the yeast *Pah* protein, mammalian lipin proteins had intrinsic PAP activity, answering this enduring biological question [3]. Given a number of differences in the regulation of yeast and mammalian lipin proteins, we have elected to focus this review on the mammalian lipins.

The cloning of mammalian lipin genes resulted from another longstanding project to identify the spontaneous mutation leading to the phenotype of fatty liver dystrophic (*fld*) mice [6]. In mammals, three genes (*Lpin1, Lpin2, Lpin3*) encode lipin proteins (lipin 1, lipin 2, and lipin 3) [4,7]. Lipin family proteins exhibit distinct tissue-specific expression patterns [7]. Lipin 1 protein is enriched in adipocytes, striated muscle, and liver. Lipin 2 protein is liver-enriched and also expressed well in the intestine and central nervous system whereas lipin 3 is expressed in intestine and fat. Predictably, *fld* mice exhibit very low levels of PAP activity in most tissues where only lipin 1 is highly expressed (adipose tissue and striated muscle), but have significant PAP activity in liver, intestine, and other organs where lipin 2 is present [7,8,9]. While germline double deletion of lipin 1 and 3 or lipin 2 and 3 is tolerated in mice, the loss of lipin 1 and 2 is embryonic lethal [10], which is also consistent with functional redundancy of lipin 1 and 2, at least in mice. The importance of lipin 2 in human physiology is also demonstrated by the observation that mutations in lipin 2 cause Majeed’s syndrome, an inflammatory syndrome of osteomyelitis [11]; the mechanistic basis for which is poorly understood. 

## 2. Lipin Protein Structure and Regulation

**Figure 2:**
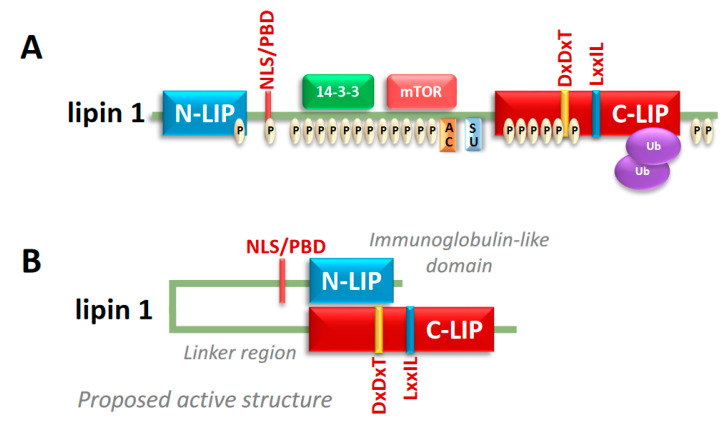
Lipin 1 structure and posttranslational modifications. (**A**) The lipin 1 protein contains several serine and threonine phosphorylation sites (P). Additionally, lipin 1 is acetylated (AC), sumoylated (SU), and ubiquitinated (Ub). Lipin 1 contains highly conserved N-terminal lipin (N-LIP) and C-terminal lipin (C-LIP) domains. The nuclear localization signal (NLS) is within the poly basic domain (PBD). The haloacid dehalogenase domain (DxDxT) is the catalytic motif and the LxxIL motif are contained within the C-LIP domain. (**B**) Recent crystal structure data suggests the N-LIP and C-LIP domains, which are separated by a linker region, interact to form an immunoglobulin-like domain in the native state.

Lipins are soluble proteins with conserved N- and C-terminal domains. A canonical haloacid dehalogenase catalytic site is contained in the C-terminal domain and N-terminal amphipathic helices and a polybasic domain facilitate membrane interaction [4,9] (Figure 2A). Recent crystallization studies have suggested that these conserved termini interact to form an immunoglobulin-like domain that is enabled by the variable regions in the middle of the protein [12] (Figure 2B). There is also evidence that lipin proteins form hetero- and homo-oligomers in their native state [13], although the importance of oligomer formation is still unclear. Atomic force microscopy imaging also suggested that lipin multimers may form circular structures or larger symmetrical particles [14]. Lipin proteins contain long stretches of basic amino acids (polybasic domain) that may be involved in promoting membrane localization by electrostatic interaction and also serve as a nuclear localization sequence [15,16] (Figure 2A). In the nucleus, lipin 1 interacts with DNA bound transcription factors to regulate their activity [17]. Lipin 1 has been shown to coactivate a number of nuclear receptors that regulate fatty acid metabolism [17], but can also act in a repressive manner on other transcription factors [18]. Since this activity is independent of PAP activity, the transcription regulatory function of lipin proteins will not be discussed in this review. 

Lipin activity seems to be controlled at several regulatory levels, though the control of the lipin 1 isoform is best understood compared to lipin 2 and 3. Transcription of the *Lpin1* gene is dynamically regulated in response to a variety of metabolic stimuli and disease states [17], but a great deal of lipin 1 activity is regulated post-translationally. Lipin 1 is a phospho-protein that is a target of the mechanistic target of rapamycin complex 1 (mTORC1) signaling pathway [9,19] (Figure 2A). Hyper-phosphorylation of serine/threonine residues of lipin 1 by mTORC1 drives its localization to the cytosol and away from the membrane and nuclear compartments [9,20]. Since PA is an insoluble lipid and embedded in cellular membranes, lipin 1 phosphorylation likely has the effect of reducing conversion of PA to DAG without affecting intrinsic PAP activity. mTORC1 is an important nutrient-sensing kinase and is downstream of the insulin receptor signaling cascade; thus, linking nutritional status to lipin 1 activity. In addition, the modification of lysine residues in lipin 1 by sumoylation [21], acetylation [22], and ubiquitination [23] can regulate lipin 1 localization and degradation, though it is unknown whether there is interplay among these various lysine modifications to modulate lipin 1 stability and activity (Figure 2A). Although less is known about the regulation of lipin 2, some studies have shown it is regulated both transcriptionally and translationally [24] and also post-translationally via phosphorylation [25]. Very little is known about the regulated expression and control of lipin 3 activity. The modulation of lipin expression and activity at multiple regulatory levels allows the cell to tightly control the activity of this enzyme.

## 3. Phosphatidic Acid and Diacylglycerol as Regulators of Signaling Pathways

For many years now, PA and DAG have been recognized as important regulators of intracellular signaling pathways and membrane biophysical properties as recently reviewed [26,27]. There are several enzymatic reactions that synthesize or catabolize these intermediates. For example, like PAP proteins, Mg^2+^-independent lipid phosphate phosphohydrolases (LPPs) dephosphorylate PA into DAG [28]. While LPP activity is important in controlling PA- and DAG-mediated signaling, LPP activity occurs primarily at the plasma membrane. LPPs also dephosphorylate LPA, ceramide-1-phosphate, and sphingosine-1-phosphate [28]. Although many of these signaling pathways are parallel to those affected by lipin expression [29], we focus our attention to DAG- and PA-responsive pathways shown to be specifically regulated by lipin-mediated PAP activity. 

Many of the effects ascribed to PA or DAG have been mechanistically demonstrated. However, it is important to note the near impossibility of modulating the abundance of one lipid without affecting levels of other related lipids. For example, PA can be rapidly converted to lysophosphatidic acid (LPA) by phospholipase A family lipases and the addition of high amounts of PA to cells in culture will likely alter abundance of LPA as well (Figure 1). Thus, caution should be taken in interpreting such results.

**Figure 3:**
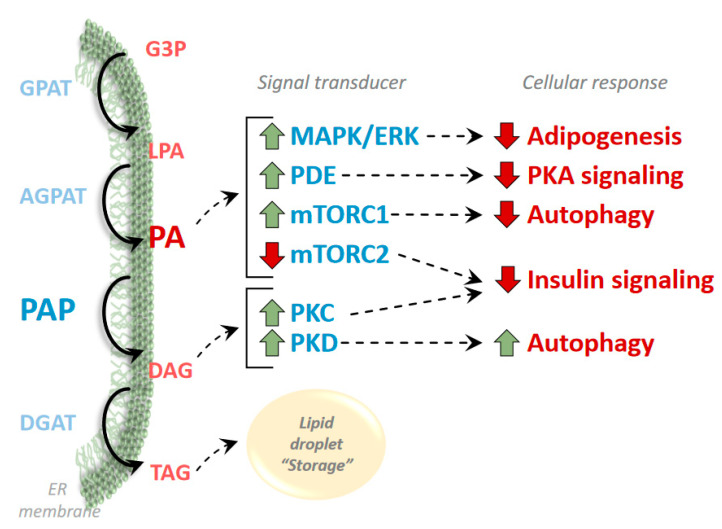
PAP derived phosphatidic acid activates several signaling cascades. Phosphatidic acid (PA) and diacylglycerol (DAG) synthesized from PAP activity effects several signaling modules involved in metabolism, autophagy, and differentiation. Enzyme abbreviations not listed in Figure 1 in **blue**: Mitogen Activated Protein Kinase (MAPK), Extracellular Regulated Kinase (ERK), phosphodiesterase (PDE), mechanistic Target of Rapamycin Complex 1 & 2 (mTORC1, mTORC2), Protein Kinase C (PKC), Protein Kinase D (PKD).

The mTOR signaling cascade is one of the most prominent kinases regulated by PA abundance [30,31] (Figure 3). As discussed above, mTOR is a nutrient responsive kinase that forms at least two distinct complexes of accessory proteins that regulate a multitude of downstream targets [32]. mTORC1 regulates protein synthesis, autophagy, mitochondrial metabolism and transcription of enzymes involved in lipid synthesis, whereas mTOR complex 2 (mTORC2) negatively regulates insulin signaling, controls cell stress response, apoptosis and cytoskeleton organization [32]. mTORC1 directly interacts with PA and this interaction allosterically activates mTORC1 to initiate a mitogenic response [33]. PA activation of mTOR, appears to have similar effects as insulin stimulation in myocytes [34]; yet, PA has also been shown to inhibit insulin signaling and is anti-mitogenic in adipocytes [35]. Work in lipin 1-deficient mice has demonstrated that mTORC1 activity is chronically elevated in some tissues [36]. mTORC1 has important negative regulatory effects on autophagy and mice or cells lacking lipin proteins exhibit general defects in autophagy [37,38]. Interestingly, PA accumulation seems to inhibit the activity of the mTORC2 signaling cascade [39]. In hepatocytes, lipin 1 knockdown leading to PA accumulation was associated with reduced mTORC2 activity and insulin resistance [39].

mTOR signaling also enhances the activity of phosphodiesterase (PDE) enzymes that degrade cAMP to control the activity of cAMP-responsive Protein Kinase A [40,41]. Additionally, PA directly activates PDE4 via allosteric interaction [42]. These dual mechanisms have been linked to impaired PKA signaling in lipin 1-deficient tissues including adipose tissue [43] and heart [36,44].

PA has also been shown to activate the extracellular signal-regulated kinase (ERK) Mitogen-Activated Protein Kinase (MAPK) signaling cascades [45,46,47] (Figure 3). This was first demonstrated in Schwann cells and is involved in the peripheral nerve demyelination that occurs in *fld* mice [46]. Regulation of ERK signaling may also be involved in the effects of lipin 1 on myocyte and adipocyte differentiation [45,47].

PA has also been shown to have effects on gene transcription by multiple mechanisms in cultured cells. Accumulation of some species of PA is linked to inhibition of peroxisome proliferator-activated receptor (PPAR) activity likely by effects on signaling pathways as well as cyclic phosphatidic acid possibly acting as an antagonistic ligand for this nuclear receptor [48]. It is possible that this plays a role in the regulation of adipocyte differentiation by this transcription factor and explains why nuclear-localized PAP activity is required for the induction of the adipogenic program in these cells [16]. Other work has suggested that the lipin-mediated remodeling of PA to DAG in the nuclear membrane by lipin 1 may also regulate gene expression by affecting chromatin structure and function [20].

In addition, the product of lipin 1 PAP activity, DAG, is also a significant regulator of signaling cascades including Protein Kinase C (PKC) and Protein Kinase D (PKD) (Figure 3). Activation of PKC isoforms by DAG accumulation in insulin-sensitive tissues has been linked to insulin resistance in obesity [49], and in mouse liver, lipin 1 mediated DAG production led to insulin resistance via activation of PKCε [50]. Additionally, in keratinocytes, lipin deficiency led to reduced activation of PKCα and affected the differentiation program of these cells [51]. Moreover, loss of lipin 1 and subsequent reductions in DAG levels in skeletal myocytes have been linked to inhibition of PKD activity, which led to impairments in autophagic flux and skeletal myopathy in *fld* mice [38].

Below we will detail the known connections between lipin and PA and its impact on signaling and metabolism in four tissue types. In order to focus this review, we will not discuss important findings in other types of cells and apologize for any oversights or unintentional exclusions. 

## 4. Adipose Tissue

Mutations in lipin 1 lead to the marked lipodystrophic phenotype of *fld* mice [4], which is consistent with the effects of mutation or knockout of other enzymes involved in triglyceride synthesis also resulting in lipodystrophy [52]. This is somewhat predictable given that lipin 1 is highly expressed in adipocytes and the role of its enzymatic activity in triglyceride storage. However, in addition to an inability to store fat, lipin 1-deficient adipocytes also fail to induce the expression of canonical genes of the adipogenic program in vitro in response to a differentiation cocktail of hormones [53,54]. Accumulation of PA may explain this observation as this lipid has been shown to activate anti-adipogenic signaling, such as the ERK-MAPK pathway [53,55], and PA inhibition of differentiation is rescued by blocking ERK signaling in 3T3-L1 cells [56]. Complementation studies have also shown that both PAP activity and nuclear localization of lipin 1 are required for adipogenesis to occur in vitro, raising the possibility that this activity is required in the nucleus to induce adipogenesis [16]. This could also fit with lipin 1 transcriptional regulatory function enhancing activity of PPARγ [57], a crucial regulator of adipogenesis. In mice, knockout of lipin 3 slightly reduces PAP activity in white adipose tissue, but does not seem to affect adiposity and lipin 3 seems insufficient to compensate when lipin 1 is absent [58]. On the other hand, humans with mutations in the gene encoding lipin 1 (*LPIN1*) exhibit no defects in adipogenesis or reduction in adiposity [26,59], likely suggesting that other members of the lipin family or other PA phosphohydrolases can compensate [60].

Conditional knockout of lipin 1 after adipocyte differentiation has begun in mice has very mild effects on adiposity on a standard diet [53,61]. Fat-specific lipin 1 knockout mice have somewhat smaller fat pads on a standard diet, but a high fat diet produces a robust phenotype and fat-specific lipin 1 knockouts are highly resistant to diet-induced obesity [53]. Despite a lean phenotype, these mice are more susceptible to insulin resistance on a high fat diet likely due to accumulation of ectopic lipid in other tissues [53]. This observation is interesting in light of translational studies showing that adipose tissue lipin 1 expression in humans with obesity correlates well with insulin sensitivity. Specifically, patients with high adipose lipin 1 expression exhibit greater insulin sensitivity in skeletal muscle and liver [62,63]. Consistent with this, lipin 1 overexpression in mouse adipose tissue promotes an obese, but insulin sensitive phenotype [54]. Though PA has not been linked to this systemic effect on insulin sensitivity per se, it is possible that this lipid or other related lipids may be involved in inter-organ communication that leads to insulin resistance and that appropriate sequestration of these lipids in adipocytes protects other tissues from lipotoxicity.

Fat-specific lipin 1 knockout mice also exhibit marked reductions in Protein Kinase A (PKA) signaling that result in impaired basal and stimulated lipolysis [43]. This was due to accumulation of PA, since other methods to increase PA abundance also impaired PKA activity. Mechanistically, PA suppressed PKA activity by a two pronged mechanism involving a direct interaction with phosphodiesterase 4 (PDE4) and by activating mTOR signaling to enhance PDE activity and reduce cAMP [43]. Interestingly, it has long been known that β-adrenergic agonists increase PAP activity [64] and stimulate lipin 1 trafficking to its active site at the membrane [9]. While counterintuitive, given the role of lipin 1 in fat storage, it is possible that this effect is a mechanism to amplify PKA signaling in response to β-agonists in adipocytes. Conversely, when PA is abundant, triglyceride synthesis is favored and lipolysis is inhibited. Though first observed in mouse adipocytes, this effect on PKA activity is also observed in lipin 1-deficient mouse liver [43] and heart [36,44] and lipin 1 abundance in adipose tissue of humans with obesity is inversely correlated with basal lipolytic rates [43]. Thus, in adipose tissue, lipin 1 plays important roles in regulating fat storage and retention by regulating both triglyceride synthesis and lipolysis.

## 5. Skeletal Muscle

Rare mutations in the *LPIN1* gene in humans are associated with a syndrome of acute, recurrent rhabdomyolysis that usually manifests in early childhood [59,65,66,67,68]. Rhabdomyolysis is an acute syndrome of skeletal muscle injury resulting in the release of intracellular metabolites and proteins, including creatine kinase and myoglobin, into the systemic circulation that can result in death from renal, cardiac, or hematologic dysfunction. Although there are many common acquired causes of acute rhabdomyolysis in children and adults, inborn errors in intermediary metabolism are often to blame in idiopathic cases, especially in children.

To decipher the mechanisms by which loss of PAP activity leads to myocyte injury, investigators have used a variety of mouse and cell culture models. It should be noted that *fld* mice [38] or mice with muscle-specific lipin 1 deletion [37,69] exhibit a chronic and active myopathic phenotype that is not a phenocopy of the acute syndrome in humans. The myopathy is characterized by myocyte necrosis, myofibrils with central nuclei indicative of regeneration, and eventually development of fibrotic lesions. Damaged mitochondria with impaired oxidative capacity accumulate in skeletal myocytes from lipin 1-deficient mice due to impaired mitochondrial turnover through the process of mitochondrial autophagy (mitophagy) [37,38]. The phenotype of *fld* mice can be rescued by transgenic muscle specific overexpression of lipin 1 [38], which together with the skeletal muscle-specific knockout models indicate a myocyte intrinsic effect. 

Loss of lipin 1 in skeletal muscle leads to very low PAP activity in muscle and both the constitutive and muscle-specific knockout of lipin 1 models all exhibit accumulation of PA and impairments in autophagy. However, the mechanistic explanations for impaired autophagy and muscle pathologic remodeling may vary upon the model used. For instance, whereas muscle-specific lipin knockouts actually exhibit increased muscle DAG content [37,69], *fld* mice exhibit depletion of DAG [38], suggesting that lipodystrophy of *fld* mice affects muscle lipid content. In *fld* mice and cells, DAG depletion leads to impaired PKD activation, which leads to defective autophagy [38] and may also affect myocyte differentiation via regulation of transcription factors that regulate developmental processes [70]. Since DAG actually accumulates in muscle of mice with conditional deletion of lipin 1 the PKD mechanism does not seem to apply to this model. Indeed, muscle-specific *LPIN1* knockout mice exhibit signs of lipotoxic and sarcoplasmic reticular stress and treatment with agents that enhance fat oxidation or chemical chaperones to alleviate stress can attenuate myopathy in these mice [69]. The mechanistic basis for myopathic remodeling in these mice and human patients with *LPIN1* mutations and optimal treatment approaches will require further study.

## 6. Cardiac Muscle

Much of the literature regarding patients with *LPIN1* mutations has focused on the skeletal muscle manifestation of the disease and less is known about the effects on cardiac myocytes, despite abundant expression of lipin 1 in the myocardium. Recently, it was shown that patients with *LPIN1* mutations have increased cardiac triglyceride accumulation and some patients exhibited a defects in cardiac function when challenged with exercise [71]. This may suggest that diminished mitochondrial oxidative capacity under exercise conditions impairs cardiac function with energetic challenge.

The role of lipins in regulating cardiac metabolism and function has been more extensively studied in mice. Lipin 1 and 3 seem to be expressed in the myocardium, but lipin 2 is not [8]. Despite expressing lipin 3 in heart, *fld* mice exhibit very little cardiac PAP activity and increased cardiac PA [8]. When isolated hearts from *fld* mice were perfused with ^3^H-oleate, the radiolabeled fatty acid was more enriched in glycerophospholipids (PA, PI, PS, etc.), but cardiac triglyceride levels were not affected [36]. Hearts from *fld* mice actually exhibited cardiac triglyceride accumulation during prolonged fasting despite decreased PAP activity [72]. *Fld* hearts also exhibit mild cardiac dysfunction, but do not exhibit cardiac myocyte death or signs of fibrosis. The reasons for the different outcomes in lipin 1-deficient skeletal and cardiac myocytes is not yet clear. We have recently developed mice with cardiac-specific *LPIN1* deficiency (manuscript submitted) and like *fld* mice, cardiac-specific deletion of lipin 1 does not lead to myocyte dropout or development of myopathic remodeling [44]. However, the cardiac lipin 1 knockouts also exhibit accumulation of PA, diminished mitochondrial respiration potentially due to reduced cardiolipin content, and mild contractile dysfunction when challenged with dobutamine. Interestingly, lipin 1 expression and PAP activity are diminished and PA abundance is increased in acquired forms of heart failure in mice [8]. It is unknown whether loss of lipin 1 and the accumulation of PA may contribute to the impairments in contractile dysfunction in these models.

Loss of lipin 1 in myocardium has been shown to have several signaling effects, including activation of mTOR signaling [36]. Whereas mTOR activation is usually linked to cardiac hypertrophy, hearts from *fld* mice are actually smaller than control hearts. It is possible that the activation of mTOR is an adaptation to regulate cardiac energy metabolism. Kok and colleagues also noted reduced phosphorylation of hormone sensitive lipase [36] in *fld* mouse hearts, which is consistent with impairments in PKA activity. Indeed, cardiac-specific lipin 1 knockout also leads to impairments in PKA activity, especially in the context of β-adrenergic stimulation [44]. This likely explains the impairment in contractility observed in response to dobutamine. Further work to investigate whether these observations translate to humans and to better characterize the cardiac phenotype of patients with *LPIN1* mutations is needed.

## 7. Liver

Unlike striated muscle and adipose tissue, the liver highly expresses both lipin 1 and lipin 2. Although lipin 2 is more abundant than lipin 1 in normal mouse liver [7], hepatic lipin 1 expression is highly induced by fasting, diabetes, glucocorticoid administration [17], and experimental alcoholic fatty liver disease [73,74]. Lipin 2 mRNA is also induced in liver by fasting and diabetes, but lipin 1 and lipin 2 are under the control of different regulatory pathways [24]. These physiologic contexts with increased lipin expression were shown many years ago to be associated with increased hepatic PAP activity [75]. 

The high expression of both proteins in liver often leads to a great deal of compensation and there are limited effects of deleting only one lipin family member. For instance, neonatal *fld* mice exhibit an overt fatty liver phenotype [6,76], which is at odds with the role of lipin 1 as a PAP enzyme involved in triglyceride synthesis. However, we now know that lipin 2 protein abundance is markedly increased in *fld* liver [24] and that the fatty liver in this model is largely driven by loss of lipin 1 in adipose tissue driving a lipodystrophic phenotype [43,68]. Conversely, knockout of lipin 2 leads to increased lipin 1 protein abundance in liver and does not affect hepatic triglyceride levels [77]. Acute knockdown of lipin proteins circumvents some of these compensatory effects and has revealed pathophysiological roles for lipin 1 and 2 in mouse models of fatty liver disease.

In many models of obesity-related related fatty liver disease, lipin 1 expression is increased [17,50]. However, not all obese mouse models exhibit an induction in lipin 1 [78] and lipin 1 seems to be decreased in human subjects with obesity and hepatic steatosis [62]. Interestingly, liver-specific deletion of lipin 1 does not prevent hepatic TAG accumulation in fasted mice. Liver lipin 1 knockout mice fed a diet enriched in fat, fructose, and cholesterol were also not protected from triglyceride and DAG accumulation or insulin resistance [77], suggesting that lipin 2 may be able to compensate for loss of lipin 1. On the other hand, Ryu et al. showed that acute RNAi-mediated lipin 1 knockdown attenuated hepatic steatosis and improved insulin-stimulated AKT activation in mouse liver and primary mouse hepatocytes [50]. Mechanistically, activation of lipin 1 can increase cellular DAG; thereby activating PKCε and driving insulin resistance [50]. Similarly, activation of lipin 2 in fatty liver or by ER stress was also shown to cause insulin resistance by this mechanism [79]. However, other work in hepatocytes has suggested that PA induces insulin resistance by suppressing mTORC2 and that lipin 1 overexpression actually attenuated insulin resistance [39]. Furthermore, in a genetic model of obesity, the UCP-DTA mouse, lipin 1 expression was decreased and lipin 1 overexpression improved insulin signaling and glucose tolerance [78]. Thus, the role of lipin 1 in hepatic insulin resistance remains controversial. 

It has long been known that PAP activity is induced in rodent models of alcoholic fatty liver disease (AFLD) [80] and that this coincides with a marked induction in lipin [73,74]. Surprisingly, liver-specific lipin1-KO did not attenuate, but actually exacerbated triglyceride accumulation and liver injury in a model of alcohol feeding, likely due to reduced fatty acid oxidation and triglyceride secretion [73]. This suggests that the induction of lipin 1 in hepatocytes in AFLD may actually play an adaptive or protective role by mechanisms that are still not completely clear. Interestingly, deletion of lipin 1 in myeloid cells markedly attenuated hepatic inflammation while concomitantly exacerbating hepatic steatosis in another model of AFLD [81]. This effect was attributable to altered secretion of adipokines like fibroblast growth factor 15 and adiponectin. Thus, the effects of lipin 1 PAP activity in myeloid cells may impact systemic inflammation and metabolism by altering interorgan endocrine signaling pathways. 

Recently, it was demonstrated that lipin 1 and 2 expression is decreased after experimental overdose with acetaminophen (APAP) in mice coincident with a marked increase in liver and plasma PA concentrations [82]. It is possible that lipin deactivation and PA accumulation after APAP overdose is an adaptive mechanism to stimulate the hepatocyte proliferative response to regenerate liver tissue. The mechanisms by which this occurs are still emerging. However, activation of mTOR signaling after APAP treatment seems to precede the accumulation of PA, suggesting that PA is not the trigger for this response. It is possible that PA is activating other mitogenic signaling pathways. 

## 8. Summary 

In conclusion, lipin-mediated PAP activity plays important and pleiotropic roles in regulating lipid metabolism and cellular homeostasis via the metabolism of PA concentrations. Indeed, it may be that limiting the accumulation of this unabundant lipid to modulate signaling pathways and limit PA toxicity could be considered the primary function of this family of phosphohydrolases. The robust phenotypes of mice and humans with lipin deficiency underscores the important roles that lipin 1 and 2 play in regulating adipocyte differentiation, myocyte homeostasis, and whole-body metabolism. Future work will almost certainly define more important physiologic and pathophysiologic roles for lipin proteins in modulating metabolism and signaling.
